# Comparative examination of YOLO models for classification and utilization in nipple injuries

**DOI:** 10.3389/fmed.2026.1776068

**Published:** 2026-10-21

**Authors:** Ruyi Zhang, Yang Shu, Sijie Ding, Yongmei Gao

**Affiliations:** 1 School of Nursing, Jilin University, Changchun, Jilin, China; 2 The First Hospital of Jilin University, Changchun, Jilin, China

**Keywords:** classification, deep learning, medical image analysis, nipple injury, object detection, YOLO

## Abstract

**Introduction:**

Nipple injury is a significant health issue that affects breastfeeding rates globally. Early diagnosis is essential for successfully preventing nipple injury and enhancing breastfeeding rates. The current approach of detecting nipple injuries mostly relies on the subjective assessments of healthcare practitioners. This approach has many disadvantages, including significant subjectivity, limited efficiency, and insufficient diagnostic consistency. This project aims to create an automated diagnosis technique that is objective and successful. YOLO served as the basis for developing an automated approach for detecting and classifying nipple injuries.

**Methods:**

The preliminary dataset included 613 images of nipple injuries collected from nursing moms in the obstetrics department of the First Hospital of Jilin University between March 2024 and July 2025. The total was increased to 2,452 photos by the use of data augmentation techniques. Utilizing this dataset, we conducted training and assessment on six distinct iterations of the YOLO model: YOLOX , PICOdet, YOLOv5, RTDETR, YOLOv8, and YOLOv11.

**Results and Discussion:**

The performance studies indicated that YOLOv11 achieved a detection accuracy of 0.85 for nipple injuries, markedly above the performance of other models (PICOdet: 0.78; YOLOv5: 0.75; RTDETR: 0.68; YOLOv8: 0.72; YOLOX: 0.74). Moreover, YOLOv11 exhibited superiority in critical measures, including Precision (P: 0.85) and recall (R: 0.82). To strengthen comparative completeness, representative two stage detectors (Faster R-CNN and Cascade R-CNN) were further included as baselines, and YOLOv11 remained superior under a unified COCO-style IoU evaluation protocol.

## 1 Introduction

Breastfeeding has long been a critical issue in worldwide public health. Breast milk, the most natural and optimal nourishment for babies, has many benefits. It offers substantial advantages for newborn nutrition, growth, and development, while also enhancing mother health, alleviating economic burdens and social expenses for families, and mitigating the environmental effects associated with breast milk. Consequently, it has significant value for individuals, families, and society at large. The Global Strategy for Infant and Young Child Feeding, established by the World Health Organization and UNICEF, advocates for exclusive breastfeeding for the first six months of life. Subsequently, supplementary meals should be provided, and breastfeeding should continue until the kid attains the age of two or older (1). As part of China's National Program for Children's Development (2021–2030), a target has been set to attain a fifty percent exclusive breastfeeding rate for infants aged zero to six months (2).

While the majority of postpartum women choose for nursing throughout the first postnatal phase, a significant number cease breastfeeding when faced with challenges. Nipple trauma is a principal factor influencing the viability of breastfeeding, often presenting as dermal injury at the nipple apex and areola, including fissures, skin lesions, sores, erythema, edema, or blisters. A research by Page T et al. indicates that about 34% to 96% of nursing mothers encounter varied levels of nipple damage (3). Neglected nipple damage may result in exacerbated discomfort, nipple hemorrhage, inadequate breastfeeding, and complications such as mastitis and breast abscess (4). These difficulties constitute a primary cause for lactating mothers to discontinue nursing, second only to inadequate milk production in importance. Nipple trauma-induced discomfort may elevate parental tension and anxiety, thus decreasing the frequency of newborn suckling and adversely affecting breastfeeding rates. Inadequate treatment of nipple trauma may elicit adverse feelings in nursing moms, diminish their confidence in breastfeeding, and eventually result in the voluntary discontinuation of breastfeeding, thereby reducing the total breastfeeding rate. Research reveals that nursing moms experiencing nipple trauma have much decreased motivation to breastfeed compared to those without such trauma, and their likelihood of early breastfeeding cessation is thrice greater than that of mothers demonstrating strong breastfeeding commitment. Consequently, the prompt identification and management of nipple trauma has substantial clinical and public health importance.

Conventional approaches to identifying nipple injuries predominantly depend on the subjective clinical experience of healthcare personnel for evaluation and determination. These approaches categorize nipple injuries into five stages according to a grading scale: Stage 0, Stage I, Stage II, Stage III, and Stage IV. This visual diagnosis method presents numerous limitations:① High subjectivity: The absence of a systematic evaluation method results in diagnoses being primarily reliant on the experience of individual healthcare providers, which causes uneven criteria and vulnerability to subjective bias. ② Low reproducibility: Diagnostic inconsistencies may occur among evaluators, and even a single evaluator may yield variable assessments for the same case at different times, significantly compromising reliability.③ Efficiency limitations: This approach depends on manual observation and experiential judgment, rendering it time-consuming and inappropriate for large-scale screening or rapid diagnosis, thereby impeding clinical workflow efficiency. ④ Inadequate standardization: The lack of established quantitative measurements and objective evaluation tools hinders the comparison of diagnostic results across cases and the monitoring of temporal changes, obstructing uniform clinical data management. ⑤ Restricted early detection proficiency: Predominantly dependent on eye examination and palpation, it may encounter difficulties in effectively recognizing early-stage or mild nipple lesions, thereby postponing care. These limitations undermine diagnostic precision and consistency, perhaps detrimentally influencing subsequent treatment and care strategies. Consequently, investigating more objective and efficient diagnostic techniques for nipple lesions possesses considerable clinical utility.

YOLO (You Only Look Once) is a category of object identification techniques utilizing deep neural network topologies, characterized by real-time detection capabilities (5). The fundamental principle regards object detection as a single-stage regression task, wherein item categories and bounding boxes are predicted directly from input photos, facilitating efficient and precise detection while achieving near-real-time performance on common GPU hardware (6). Due to its high precision and efficiency, YOLO possesses significant clinical promise in medical contexts like nipple lesion identification, where swift and accurate categorization and localization are essential for facilitating diagnostic processes (7, 8). Prior research has utilized YOLO models to identify and assess wounds, such as pressure injuries and burns, attaining performance levels that either match or exceed those of human specialists in specific tasks, so illustrating its promise in medical picture analysis (9). Nipple lesions display distinct and highly variable morphology, size, color, and surface attributes, presenting significant challenges to the model's feature extraction and generalization skills. Variations in nipple size, shape, surface imperfections, and color may influence detection accuracy and reliability. This research thoroughly evaluates and analyzes various prominent YOLO variations, including YOLOX, PICOdet, YOLOv5, RTDETR, YOLOv8, and YOLOv11, to improve detection capacities for nipple injuries. This study utilizes various data augmentation techniques to improve models’ adaptability and resilience to real-world image fluctuations. Experimental designs involve training, evaluation, and hyperparameter optimization across various models, with the objective of uncovering their distinct strengths, shortcomings, and ideal application contexts for nipple lesion detection and staging tasks. The proven optimum models are incorporated into a mobile clinical mini-program to facilitate real-time identification of nipple lesions for nurses and patients, offering actionable evidence and resources for prompt clinical diagnosis and enhanced treatment strategies. The system's time efficiency, detection reliability, and transferability are assessed through application in real clinical settings, hence enhancing the practical deployment of this technology for early diagnosis and ongoing monitoring of nipple lesions.

## 2 Research methods

### 2.1 Research subjects

This study gathered pertinent photos from lactating mothers who experienced hospital births at the First Hospital of Jilin University and displayed signs of nipple injury during their postpartum hospitalization. A total of 613 photographs were collected from March to July 2025.

The criteria for inclusion of study volunteers were as follows: ① Women aged ≥18 years who are lactating; ② Occurrence of nipple injury during postpartum hospitalization, confirmed by independent evaluation from two experienced nurses utilizing a standardized nipple injury grading scale; ③ Ethical committee approval and written informed consent acquired from the patient. The study complied with pertinent ethical standards, ensuring that data collection, storage, processing, and analysis were executed in alignment with principles of minimization and de-identification to safeguard privacy and security.

The exclusion criteria were as follows: ① Concurrent or simultaneous diagnoses of other dermatological disorders (e.g., allergic dermatitis, psoriasis) that may impede the diagnosis of nipple injuries or undermine the accuracy of observational assessments. ② Nipple injury locations concealed by dressings, tattoos, cosmetics, or other materials obstructing objective observation, recording, or imaging of the impacted region. ③ Additional factors that may influence data quality, observational integrity, or study safe.

### 2.2 Research team

The research team, consisting of six people (refer to Table 1), was directed by the Head Nurse of the Obstetrics Department at the First Hospital of Jilin University. Team members exhibit substantial intellectual and clinical expertise, including two Master's degree holders, one Master's candidate, and three individuals with undergraduate qualifications. The core members are senior obstetric nurses possessing Registered Nurse Certification in Obstetrics (RNC-OB) and have a minimum of 20 years of substantial expertise in clinical breastfeeding management. Roles are distinctly delineated: three nurses accredited by the Chinese Nursing Association oversee breastfeeding technical guidance and project quality assurance; concurrently, two more obstetric nurses with substantial clinical expertise execute specialized intervention strategies. To ensure standardization and efficiency, all research members received comprehensive and uniform training prior to the project's initiation. The training covered breastfeeding theory, clinical operational skills, and image collection techniques. Following the training, all members passed standardized assessments to confirm their competency. This process was designed to guarantee the consistency and professional execution of the study procedures.

**Table 1:** Project team members.

Position	Age (years)	Professional title	Academic qualifications	Years of service (years)
Head Nurse	52	Chief Nurse	Undergraduate	30
Ward Nurse Manager	50	Deputy Head Nurse	postgraduate	20
Nurse	49	Senior Nurse	postgraduate	15
Nurse	36	Senior Nurse	Undergraduate	5
Nurse	25	Junior Nurse Practitioner	Postgraduate student	1

### 2.3 Image collection

Clinical nurses acquire photos of nipple injuries during standard care procedures. Following a first evaluation revealing nipple injury, nursing personnel will electronically record the affected area to guarantee the promptness and impartiality of the visual documentation. To ensure uniform and professional image quality, both the imaging apparatus and parameters have been standardized: Imaging Apparatus: ① Primarily employing the Fuji XT-4 camera (21.6 million effective pixels) in conjunction with the Fuji XF 60 mm F2.4 R Macro lens. A grey card is utilized during capture to calibrate color and reduce environmental lighting interference with color reproduction. ② Capture Mode: Consistently utilizing ‘Image Stabilization + Auto Mode’. Image stabilization reduces blurring from handheld shooting, while automatic mode offers consistent basic settings, maintaining operational uniformity. ③ Fundamental shooting parameters: All image acquisition follows standardized parameters: shutter speed of 1/60 second, ISO range of 3,600–6,000, aperture between F6 and F8, focal length of 60 mm, and white balance configured to automatic mode. The criteria for image inclusion are as follows: ① Images must be obtained by the study's investigators to guarantee uniformity in both scene and methodology; ② Images must comprehensively illustrate the nipple damage region and adhere to the study's technical specifications (e.g., resolution, shooting angle). The criteria for image exclusion are as follows: Images displaying blurriness, overexposure, or other complications that hinder the precise identification of lesion characteristics; Images featuring unaltered skin tissue or impediments such as garments or linens that limit lesion evaluation. This standardized procedure guarantees that gathered photos retain high consistency and professional quality, establishing a dependable visual data foundation for further image analysis and model training.

This study employed the Nipple damage Grading Scale to categorize all nipple damage photos into five stages, as outlined in Table 2 (10, 11). Figure 1 presents representative instances for each level, whereas Figure 2 depicts the full implementation process. Image staging was conducted by two independent evaluators, both accredited as Registered Nurse Clinicians in Obstetrics (RNC-OB) with pertinent clinical expertise to guarantee staging precision and clinical significance. The initial staging was conducted autonomously by a systematic comparison of image attributes. In instances of disagreement among assessors over the staging of a single photograph, a third assessor was promptly summoned for an impartial evaluation. The image was included in the subsequent data processing and deep learning model training dataset only when consensus was achieved among all three assessors. After validated staging, rectangular bounding box annotations were applied to photos at all five stages utilizing the Roboflow web platform. After all annotations are completed, a nurse with a minimum of ten years of clinical experience must validate the annotation labels to guarantee precision and uniformity. This procedure yields superior annotated data to facilitate future model training.

**Table 2:** Nipple trauma scoring scale1.

Grade	Affected area	Clinical presentation
Level 0	The surface of the nipple is intact	The skin is normal, with no redness, swelling, blisters, cracks, or similar issues.
Level I	The surface of the nipple is intact	Redness/erythema of the nipple, ecchymosis/bruising, oedema/swelling.
Level II	Superficial tissue injury to the nipple	Nipple abrasions, superficial fissures or cracks, compression striations, bleeding/haematoma, superficial ulceration
Level III	Nipple injury invading deep tissue	Deep fissures, blisters, deep ulcers, severe erosion.
Level IV	Extensive deep tissue damage to the nipple	Part of the nipple has been completely eroded.

**Figure 1:**
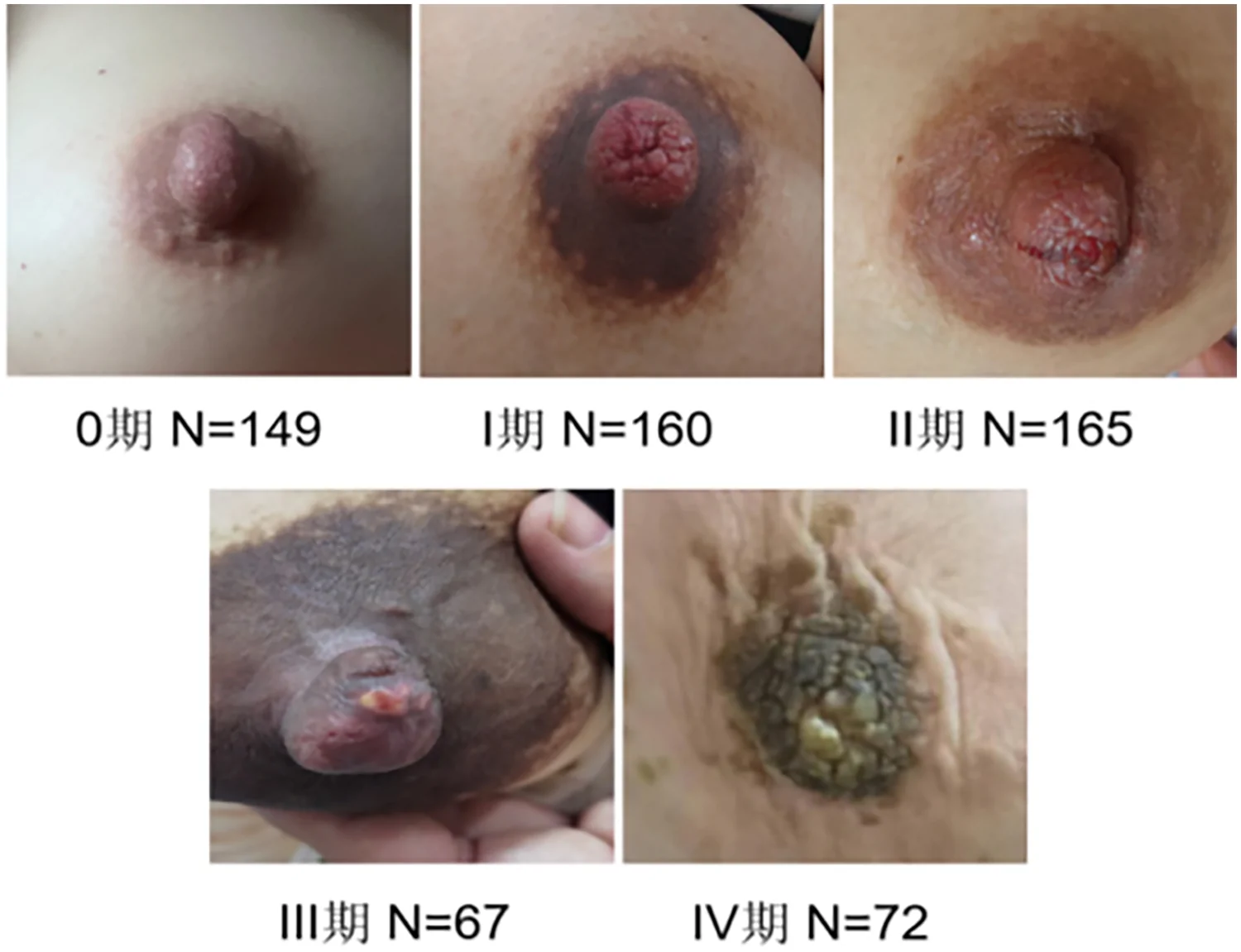
Representative images and their distribution in the dataset.

**Figure 2:**
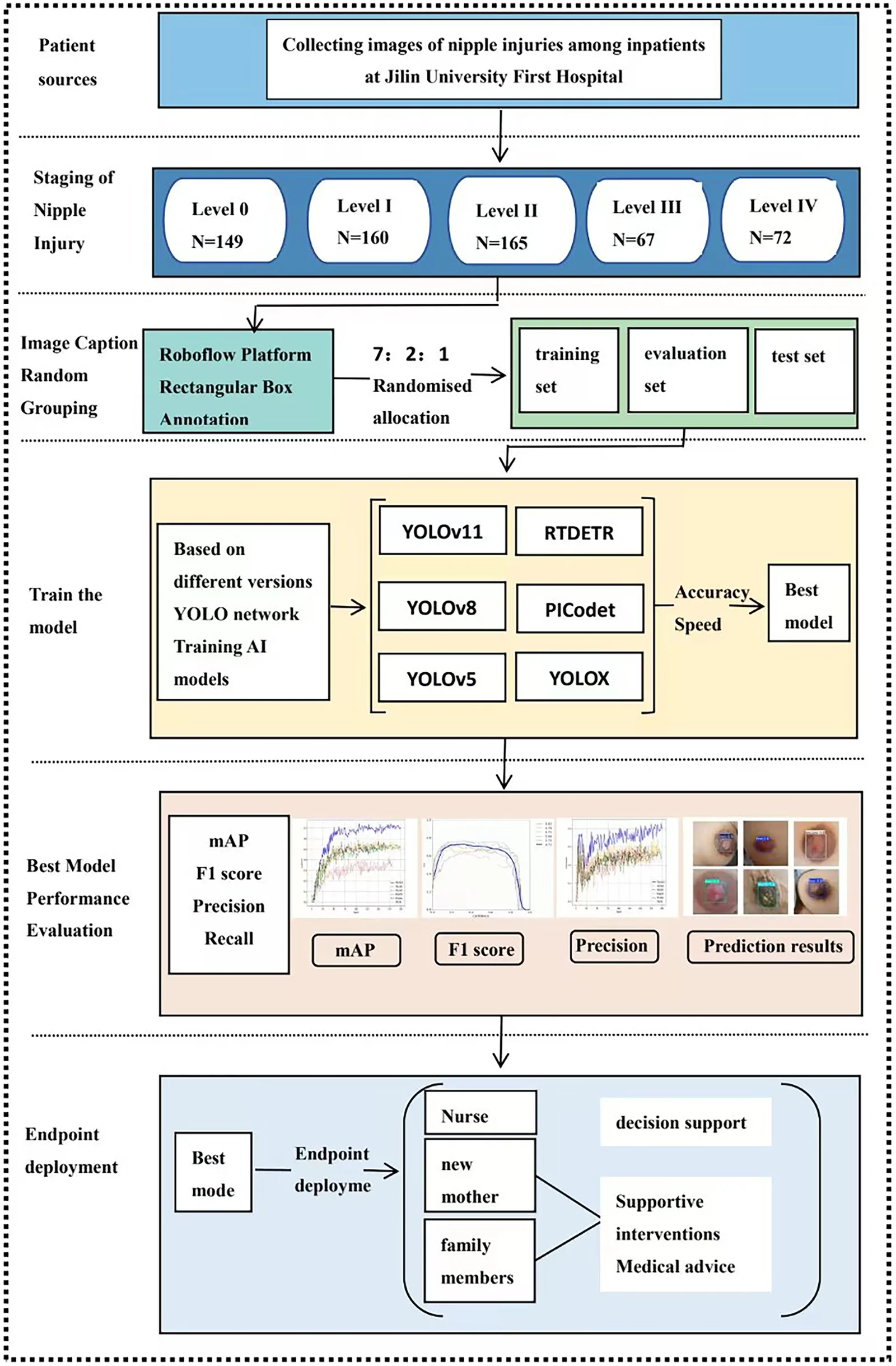
Research flowchart.

### 2.4 Data augmentation

The adequacy of sample data is a fundamental need for optimal performance of deep learning models, demonstrating a strong positive association between the two. Sufficient samples can thoroughly encompass the many attributes of nipple lesions and intricate clinical situations, so boosting the model's generalization capacity, mitigating the risk of overfitting, and bolstering robustness (10, 12, 13). This establishes a robust basis for clinical translation and implementation. The intrinsic complexity of nipple injuries, their morphological diversity, and the ambiguity of clinical imaging settings considerably limit the model's detection efficacy and precision. These limitations specifically appear over three dimensions, each aligned with distinct optimization directions: Initially, the influence of distinct nipple attributes. Substantial individual variability in nipple and areola pigmentation among nursing women may mask or complicate lesion characteristics, thereby hindering the model's accurate target selection. This interference can be alleviated by image color normalization techniques. Secondly, constraints in the fraction of lesion area. The impaired region of the nipple comprises a negligible fraction of the picture, representing a standard ’small object identification’ challenge. This significantly elevates the challenge for the algorithm in identifying the impaired region and extracting its characteristics. Algorithms for targeted tiny object augmentation are necessary to enhance the capability of local feature capture. Thirdly, interference from clinical environmental factors. External factors, including light intensity, imaging device specifications, and shooting angles, can lead to variations in image quality, thereby diminishing the model's clinical applicability. This issue can be alleviated by creating simulated clinical variant samples or implementing standardized clinical imaging protocols.

This work utilized online data augmentation approaches for model training after picture annotation to improve the accuracy of artificial intelligence models in recognizing nipple lesion photos. This technology facilitates real-time variation processing of input image data without the creation of new physical files, allowing the model to meet marginally different examples in each repeat training cycle. This method enhances the model's ability to adjust to visual fluctuations present in actual clinical situations. This solution directly tackles the intrinsic constraints of conventional manual diagnosis, which contends with image variability and is susceptible to misunderstanding due to visual bias. It thereby establishes a logical complementarity between technological optimization and the deficiencies of traditional diagnostic methods.

This study utilizes a multi-dimensional image transformation technique for data augmentation to systematically improve the model's generalization capacity and environmental adaptability. The specific measures are detailed as follows (as depicted in Figure 3): ①Image standardization and geometric augmentation: All training pictures are initially standardized to 640 × 640 pixels, then undergoing random rotation. By employing dimensional normalization and expanding morphological variety, this approach effectively reduces the hazards of model overfitting while improving classification performance for unique, unseen samples. ② Colour Space Augmentation: Employing Roboflow's inherent capabilities, random modifications are applied to HSV colour space parameters, encompassing Saturation, Value, and Contrast. This allows the model to adjust to picture inputs with differing illumination levels and color distributions found in clinical environments; ③ Noise augmentation: Random noise was added to the images. This improves the model's ability to process low-clarity and blurred image data, and further enhances its robustness to low-quality images in real clinical practice. In addition to standard geometric and colour augmentations, clinically motivated, task-specific strategies were introduced. Lesion-preserving cropping was guided by annotated bounding boxes: Stage 0–II lesions are localised to the nipple area, permitting more precise cropping, whereas Stage III–IV lesions expand to the areola and breast areas and therefore require a broader cropping scope during data processing. Local contrast enhancement (CLAHE) was applied within lesion regions to improve the visibility of early erythema and micro-fissures. Class-aware sampling was further used to increase the sampling probability of under-represented advanced-stage lesions, thereby constructing a stage-aware augmentation pipeline tailored to nipple injury detection.

**Figure 3:**
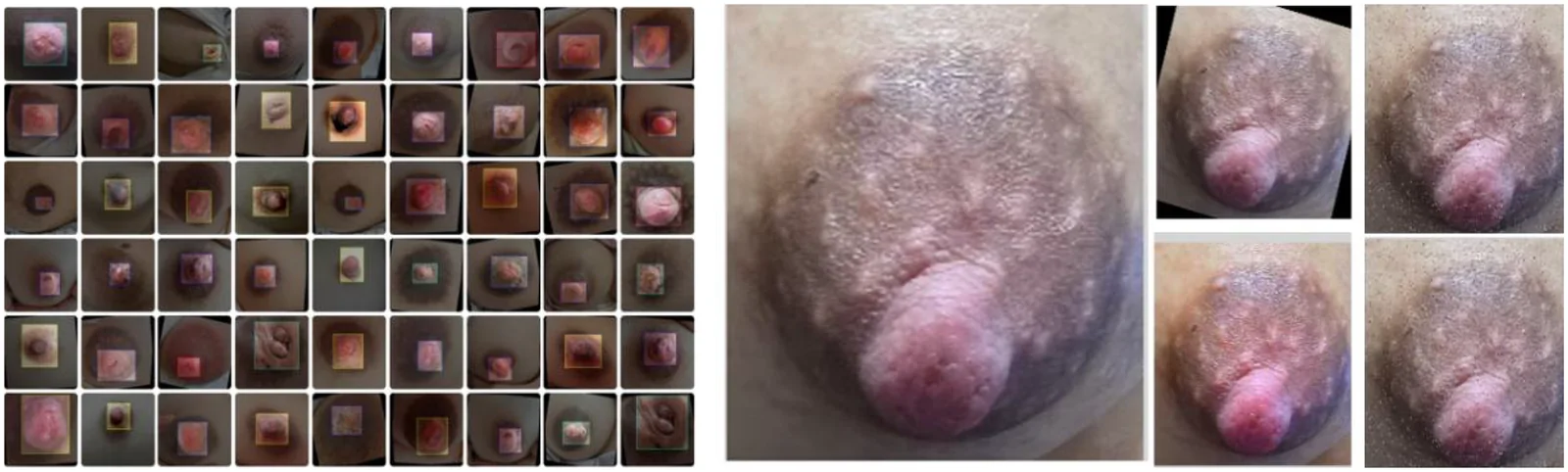
Particulars of image processing.

The previously indicated augmentation technique successfully expanded the dataset from 613 photos to 2,452 and substantially mitigated the risk of model overfitting. This improved the model's fundamental performance measures and bolstered its adaptability to many complex scenarios faced in actual clinical environments. Figure 4 depicts the dataset segmentation and the training-evaluation process. The dataset was randomly partitioned into training, validation, and test sets in a 7:2:1 ratio: 1,716 images were utilized for model training; 490 images enabled real-time validation during training to optimize model parameters; and 245 images formed an independent test set for objectively evaluating the model's generalization ability.

**Figure 4:**
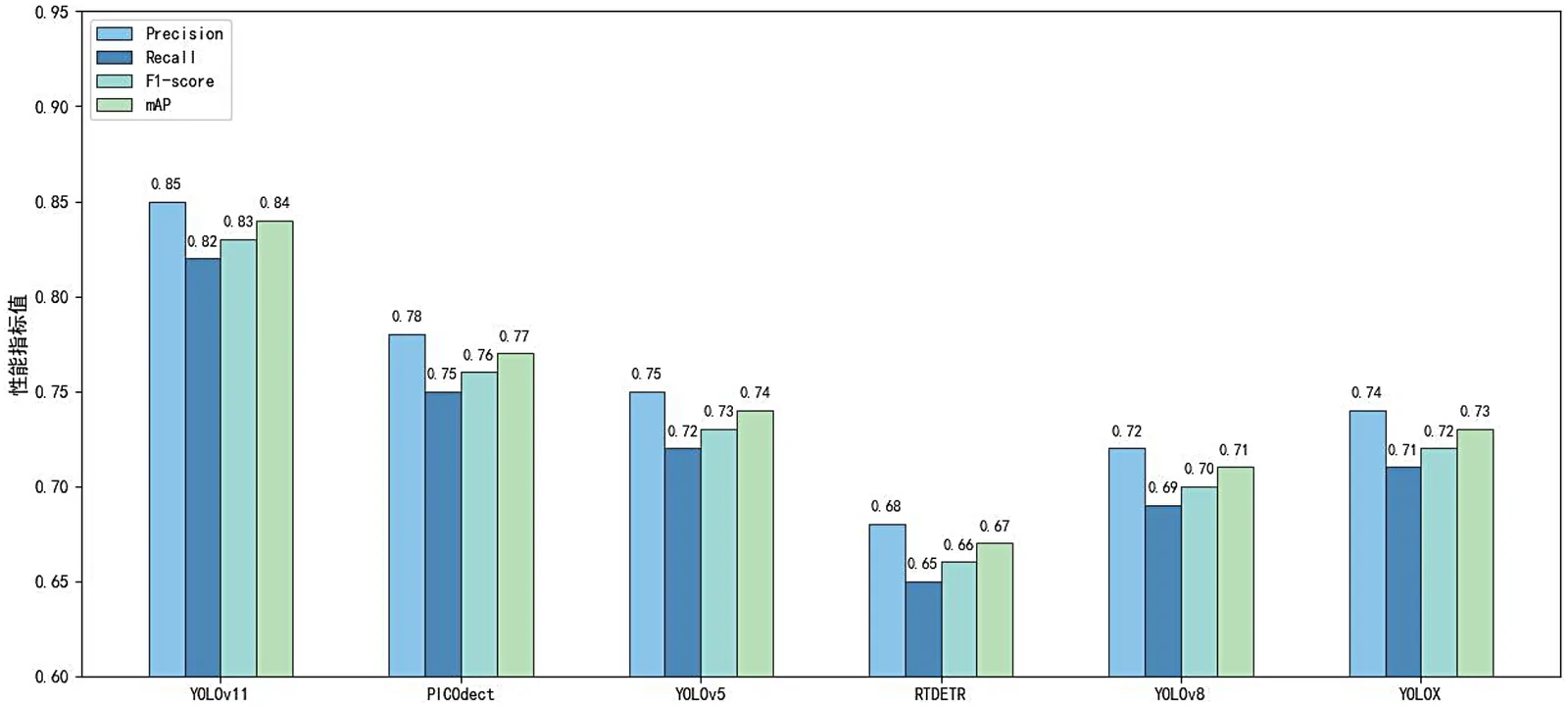
Performance metrics comparison of different target models.

The model trained on the expanded dataset exhibited substantial enhancements in accuracy, recall, and other fundamental performance parameters. The results verify the efficacy of the image augmentation strategies used in this work, highlighting their crucial role in optimizing model performance and improving clinical utility.

### 2.5 Model selection and training

#### 2.5.1 Model selection

The swift progression of artificial intelligence technologies has revealed substantial application value and transformative potential in image identification, a fundamental study area within computer vision, across several vital fields, including medical diagnostics and autonomous driving. In object detection and picture classification applications, deep learning techniques have become progressively advanced and varied. Model applicability must be assessed comprehensively by evaluating the distinct properties of the research dataset and the needs of the task. The primary objective of this research is the automated detection of nipple lesion photos. A salient feature of the data is that the lesion area comprises an exceedingly minor fraction of the image, exemplifying a conventional small object identification scenario. Therefore, model selection should concentrate predominantly on prevalent approaches in the small object identification field.

The predominant deep learning algorithms in the small object identification field currently encompass Faster R-CNN, SSD (Single Shot MultiBox Detector), the YOLO (You Only Look Once) series, and RetinaNet. Future research will do quantitative analyses based on the real attributes of this study's dataset, assessing fundamental parameters such as detection accuracy, inference speed, and model complexity, thereby determining the most suitably optimized model. The YOLO series methods are distinguished within the current object identification framework due to their significant multi-dimensional technical advantages. Initially, its advanced feature extraction architecture accurately catches intricate image features, rendering them highly effective for identifying minute lesions on the nipple; Secondly, it has exceptional scene-adaptive optimization skills, adeptly managing fluctuations in image characteristics resulting from changes in clinical imaging environments; Thirdly, its lightweight network architecture facilitates synergistic optimization of detection accuracy and inference speed; fourthly, its superior multi-task compatibility enables further expansion to derivative tasks like as nipple lesion grading and severity evaluation. These attributes provide substantial technological benefits to the YOLO series algorithms in medical picture analysis. In response to the need for comparative completeness, classic two-stage detectors (Faster R-CNN and Cascade R-CNN) were additionally included as baselines. Mask R-CNN was not adopted because the present dataset provides bounding-box annotations rather than pixel-level instance masks required by mask-based heads.

The YOLO set of algorithms especially mitigates the inherent deficiencies of conventional manual assessment approaches in the context of nipple damage identification. The traditional assessment of nipple injuries predominantly depends on visual examination and the subjective appraisal by nursing personnel, which entails several limitations: Assessment outcomes are markedly subjective, influenced by the clinician's expertise and individual interpretation; detection efficacy is inadequate, with an average duration surpassing two minutes per case, complicating the fulfillment of efficient clinical diagnosis and treatment requirements; consistency fluctuates considerably among assessors, and minor injuries are often overlooked. The technological merits of the YOLO series algorithms effectively tackle these challenges, providing an innovative technical approach for the accurate and efficient identification of nipple injuries.

Utilizing deep learning techniques to analyze and process images of nipple lesions enables the automatic detection and classification of afflicted regions. This technical solution has numerous benefits compared to conventional manual evaluation techniques: Firstly, it markedly improves the precision and efficacy of nipple lesion identification, effectively reducing the influence of human variables such as personal experience and subjective judgment on evaluation results. This significantly enhances diagnostic objectivity and reproducibility while mitigating evaluation bias due to evaluator variability. Secondly, the algorithm's rapid inference speed mitigates the time-consuming constraints of conventional detection approaches, indicating its suitability for real-time clinical application. It swiftly furnishes nursing personnel with accurate evaluation references, according to the necessity for effective diagnosis and treatment in clinical environments; This deep learning system has attained state-of-the-art performance in various traditional object detection applications. Its extensive benefits in precision, velocity, and generalizability have been rigorously confirmed, offering substantial technical evidence for its dependable use in nipple damage detection contexts.

This paper presents an automated approach for detecting nipple injuries, providing scientific and objective decision-making support for clinical therapy of such injuries. It possesses considerable practical utility and application potential for optimizing clinical care processes, improving nursing quality, and minimizing healthcare resource utilization.

#### 2.5.2 Model training

This study selected Precision (P), Recall (R), Mean Average Precision (mAP), and F1 score as essential measures to evaluate the model's performance in nipple lesion identification thoroughly. The definitions, computation methodologies, and clinical relevance of each statistic are as follows:

1. Precision (P) (14)

Precision measures the accuracy of a model's predictions, defined as the ratio of correctly classified positive samples to the total predicted positive samples by the model. The formula for calculation is:

$$P=\frac{NTP}{\left(NTP+NFP\right)}$$

In this formula, NTP signifies the quantity of true positives, referring to the nipple lesion samples accurately recognized by the model; NFP indicates the number of false positives, denoting the samples in which the model erroneously classified normal nipple regions as lesion areas. The primary therapeutic significance of this metric is its capacity to diminish the likelihood of false positives, therefore averting unwarranted clinical interventions resulting from the misclassification of normal regions as lesions and ensuring the dependability of diagnostic results.

2. Recall (R) (15)

Recall, also referred to as the precision rate, assesses a model's capacity to detect and encompass samples from the positive category. It is defined as the ratio of accurately predicted positive category samples to the total number of actual positive samples in the dataset. The equation is:

$$R=\frac{NTP}{\left(NTP+NF\right)}$$

In this formula, FN signifies the number of false negatives, indicating the count of true nipple lesion samples that the model failed to detect. In the realm of nipple lesion detection, this statistic is directly associated with the likelihood of clinical misdiagnosis: an elevated recall rate signifies the model's superior capacity to identify small or hidden lesions. This significantly diminishes the likelihood of missed diagnoses, ensures the thoroughness of clinical evaluations, and averts delays in care interventions due to diagnostic errors.

3. Mean Average Precision (mAP) (16)

Mean Average Precision denotes the arithmetic mean of the Average Precision (AP) values across all categories in a dataset. AP represents the area beneath the precision-recall (P-R) curve, functioning as an indicator of a model's detection efficacy inside a specific category. Conversely, mAP assesses a model's overall detection efficacy across all categories. The formula for its calculation is:

$$\mathrm{PmAp}=\frac{1}{N}\overset{N}{\underset{i+1}{\sum }}\;P_{AP}(i)$$

In the formula, PAP is the area beneath the P-R curve; n signifies the number of classes.mAP@0.5 represents the mean average accuracy at an Intersection over Union (IoU) threshold of 0.5, whereas mAP@0.5:0.95 indicates the mean average precision over IoU thresholds ranging from 0.5 to 0.95.

To ensure fair and reproducible cross-model comparison, a unified COCO-style evaluation protocol was applied to all models. A detection was regarded as a true positive only when the predicted category matched the ground-truth label and the Intersection over Union (IoU) between the predicted and ground-truth boxes was no lower than the specified threshold. Specifically, mAP@0.5 denotes mean Average Precision at a single IoU threshold of 0.5, whereas mAP@0.5:0.95 denotes the mean Average Precision averaged over IoU thresholds ranging from 0.5 to 0.95 with a step size of 0.05 (ten thresholds in total). The same IoU settings were used consistently for all single-stage and two-stage detectors reported in this study.

#### 2.5.3 Training parameters

To guarantee the consistency and repeatability of the studies, all single-stage models employed for the primary comparison were trained using a standardised set of hyperparameters. The precise configurations are as follows: the number of training epochs is 200, the batch size is 16, and the input picture dimensions are uniformly resized to 640 × 640. Stochastic Gradient Descent (SGD) is utilised as the optimiser, with an initial learning rate (lr0) of 0.01, a weight decay of 0.0005, and a momentum coefficient of 0.937. All single-stage models are iteratively refined under this parameter setup and attain optimal performance at 200 epochs, ensuring advantageous convergence and generalisation capability. For the supplementary two-stage baselines (Faster R-CNN and Cascade R-CNN) implemented in MMDetection, the standard 1 ×  training schedule (12 epochs) with batch size 16 was adopted, while evaluation still followed the identical COCO-style IoU protocol described above to maintain comparability.

### 2.6 Experimental platform

Model training, testing, and image data preparation were performed in a designated hardware and software environment. The experiment employed a high-performance computer featuring a graphics card with an NVIDIA RTX 4080S (16 GB VRAM) and a central processor unit (CPU) of Intel® Core™ i9-13900KF, operating at a base frequency of 3.00 GHz. This hardware arrangement sufficiently satisfies the requirements for extensive data computation and storage during deep learning model training.

The software environment for model development, training, and image processing utilizes the Python 3.8.18 programming language, the PyTorch 2.1.0 deep learning framework, and PyTorch-CUDA 11.8 acceleration tools. This arrangement facilitates highly efficient parallel computing throughout the model training process. Table 3 presents the detailed configuration parameters for the pertinent software environment.

**Table 3:** Parameters related to the experimental platform.

Hardware and software platform	Relevant parameters
Operating System	Windows10 × 64
CPU	Intel(R) Core(TM) i9-13900KF 3.00 GHz
GPU	NVIDA RTX4080S 16GB
Model Training Environment Configuration	Python 3.8.18 + Pytorch 2.1.0 + Pytorch-cuda 11.8

This study employed the Ultralytics Hub online platform (access link: https://hub.ultralytics.com) for the distribution of the optimized model to a mobile application during the model deployment phase, facilitating portable model deployment. Ultralytics Hub is an extensive deep learning service platform centered on the YOLO model series, encompassing essential activities such as data preparation, model training, performance evaluation, and multi-end deployment. The platform provides user-friendly functionality and extensive process automation, facilitating the swift and effective transformation of trained models into mobile applications. This establishes the groundwork for the clinical advancement and practical implementation of the models.

## 3 Ethical considerations

The Ethics Committee of the First Hospital of Jilin University has authorized this study, permission number 25K094-001. All operations rigorously comply with pertinent medical ethical regulations. The informed consent process is strictly upheld during implementation: all participating patients autonomously determine their participation after comprehensively comprehending the study's objectives, details, procedures, and any dangers. Participation is completely optional. Patients retained the right to decline participation or withdraw from the study at any point without experiencing discrimination or retaliation, nor would this impact their access to routine medical care or associated benefits. If participants decide to withdraw, they may contact the study team through designated means, and the team will swiftly finalize the requisite procedural procedures. Moreover, patient confidentiality is stringently safeguarded during the trial. All patient data acquired during the research will be utilized exclusively for academic analysis and model building within this study and will not be revealed to any third party. Participants will not bear any expenses related to their participation, therefore protecting their rightful rights and interests.

## 4 Model training results

A performance comparison of multiple models was conducted to assess the adaptability differences among prevalent object detection algorithms, specifically for the purpose of detecting tiny nipple lesions, utilizing a controlled variable method. The experimental findings are as follows:

To visually depict the performance discrepancies among various models in nipple lesion detection, comparison experiments were executed utilizing six prominent object detection models: YOLOv11, PICodet, YOLOv5, RTDETR, YOLOv8, and YOLOx. All models received photos uniformly sized at 640 × 640 pixels, in accordance with the standardized specifications outlined in Section 2.3 regarding data augmentation. Table 4 presents the key performance metrics for each model, and Figure 4 illustrates the results of the multi-metric comparison. Table 4 and Figure 4 clearly indicate that YOLOv11 exhibited the most superior performance among the six single-stage models, with an accuracy of 0.85, a recall of 0.82, an F1 score of 0.83, and a mean Average Precision (mAP) of 0.84. Each metric substantially surpassed the other five models. PICodet revealed the second-highest performance (accuracy 0.78, recall 0.75, F1 score 0.76, mAP 0.77); YOLOv5, YOLOx, and YOLOv8 demonstrated moderate performance levels; RTDETR registered the lowest metrics across all parameters (accuracy 0.68, recall 0.65, mAP 0.67).

**Table 4:** Performance metrics comparison of different target models.

Model	Input size	Precision	recall	F1 score	mAP
YOLOv11	640 × 640	0.85	0.82	0.83	0.84
PICOdet	640 × 640	0.78	0.75	0.76	0.77
YOLOv5	640 × 640	0.75	0.72	0.73	0.74
RTDETR	640 × 640	0.68	0.65	0.66	0.67
YOLOv8	640 × 640	0.72	0.69	0.70	0.71
YOLOx	640 × 640	0.74	0.71	0.72	0.73

To further examine whether YOLOv11 remains competitive beyond the single-stage family, representative two-stage detectors were trained and evaluated on the same nipple-injury dataset under a unified COCO-style protocol (IoU = 0.5 for mAP@0.5). Faster R-CNN and Cascade R-CNN achieved mAP@0.5 values of 0.621 and 0.470, respectively (corresponding mAP@0.5:0.95: 0.301 and 0.203). Additional MMDetection baselines yielded mAP@0.5 of 0.578 (ATSS) and 0.508 (FCOS). Although these two-stage/anchor-based models provide strong localisation capacity, YOLOv11 (mAP@0.5 = 0.84) still outperformed them overall, supporting its suitability for automated nipple-injury detection when both accuracy and deployment efficiency are considered. Accordingly, the comparative conclusion is restricted to the detectors evaluated in this study rather than an unconstrained claim of global optimality.

Figure 5 visualises the unified-protocol comparison summarised in Table 5. YOLOv11 achieves the highest mAP@0.5 among all evaluated detectors.

**Figure 5:**
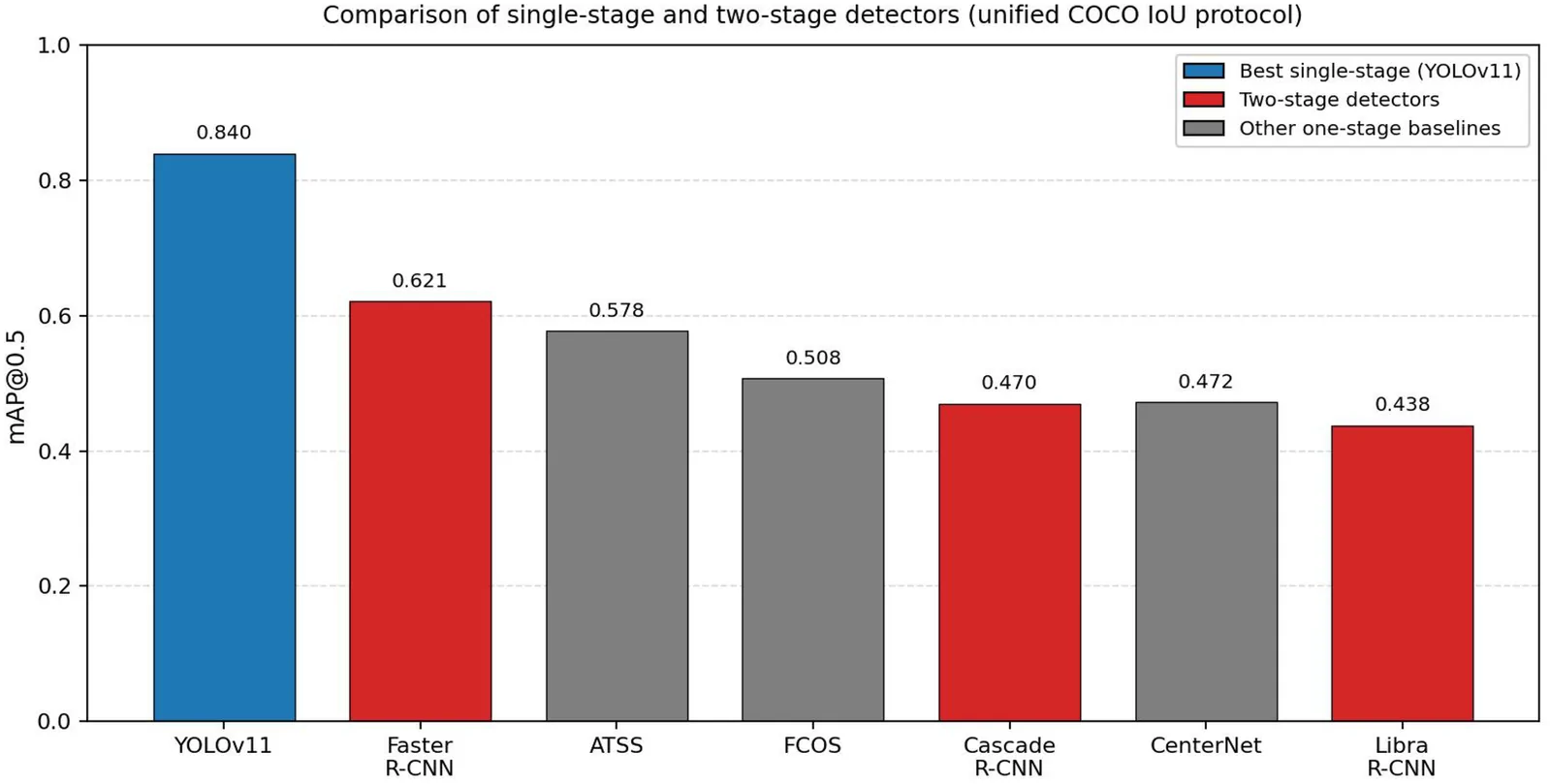
mAP@0.5 comparison of YOLOv11 against two-stage and additional baselines.

**Table 5:** Comparison of single-stage and two-stage detectors (mAP@0.5/mAP@0.5:0.95).

Model	Detector type	mAP@0.5	mAP@0.5:0.95
YOLOv11	Single-stage	0.840	—
Faster R-CNN	Two-stage	0.621	0.301
ATSS	One-stage	0.578	0.271
FCOS	One-stage	0.508	0.222
Cascade R-CNN	Two-stage	0.470	0.203
CenterNet	One-stage	0.472	0.199
Libra R-CNN	Two-stage	0.438	0.193

Figure 6 illustrates the dynamic variation curves of mAP50 (mean average precision at IoU=0.5) for six object detection models: YOLOv11, YOLOv8, YOLOv5, RTDETR, PICodet, and YOLOX, after 200 training epochs. The vertical axis represents mAP50 values, the principal metric for evaluating staging detection accuracy, while the horizontal axis illustrates the training cycle, reflecting the iterative optimization of model parameters. Higher values indicate that the model is more effective at distinguishing between stages I–IV lesions and stage 0 normal tissue. The YOLOv11 model demonstrates a rapid initial ascent rate, stabilizes at a high plateau after approximately 100 training epochs, and ultimately attains markedly superior performance relative to other benchmark models, as evidenced by results indicating that the model consistently maintains a leading mAP50 value throughout the training process. RTDETR consistently achieves a somewhat low mAP50, whereas models such as YOLOv8 and YOLOv5 exhibit far lower peak mAP50 values. The results further substantiate the YOLOv11 model's robust feature extraction and classification capabilities for nipple lesion grading tasks. It enables more precise differentiation of various levels of nipple lesions, hence enhancing the technical assistance for automated clinical assessment of these lesions.

**Figure 6:**
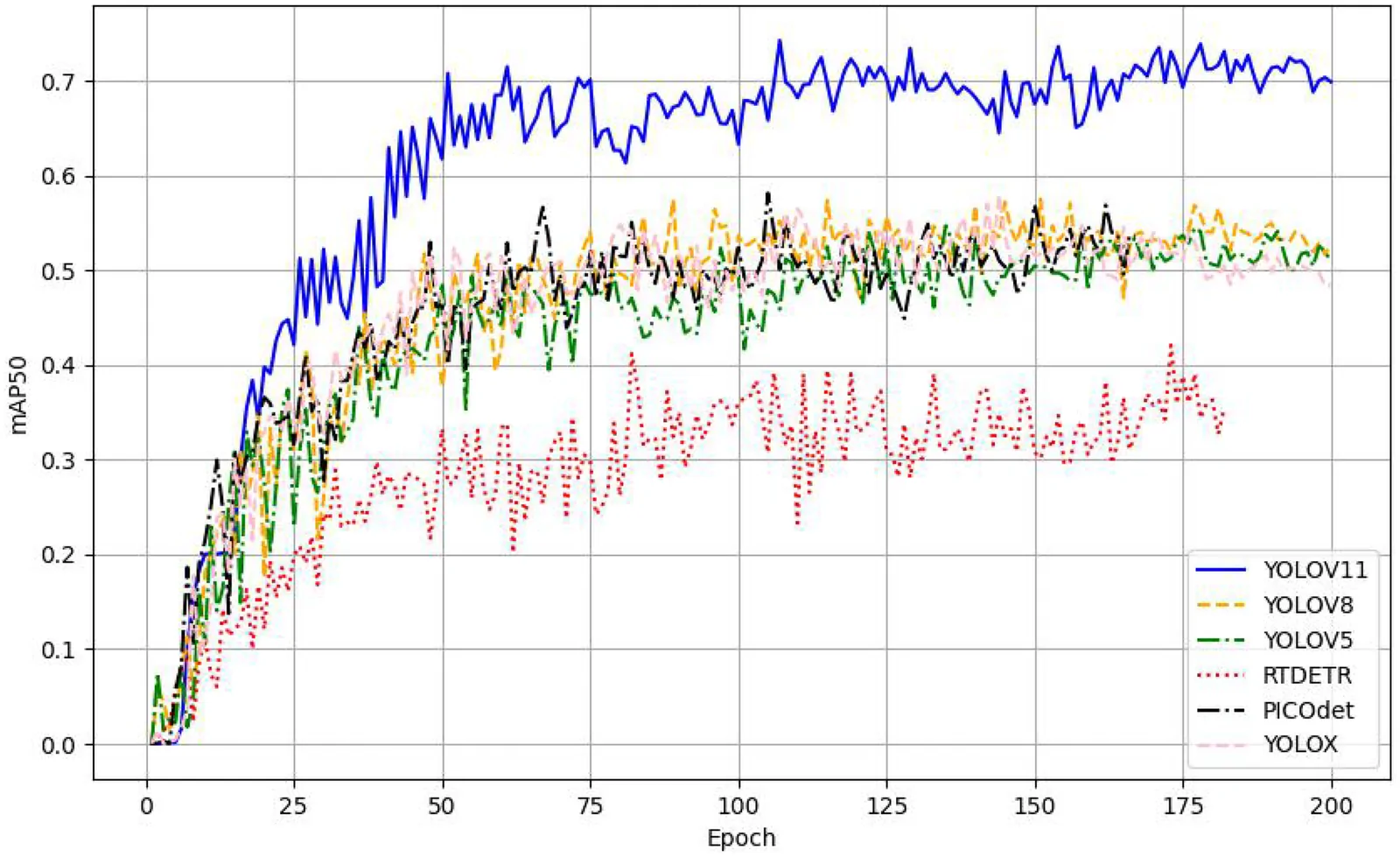
mAP50 for 200 epochs in multiple models.

This study comprehensively analyzed the dynamic characteristics of recall and accuracy for six prominent models—YOLOv11, YOLOv8, YOLOv5, RTDETR, PICodet, and YOLOX—to elucidate the evolutionary trends of training performance in different object identification models for nipple lesion detection tasks. Figures 7, 8 present the comparative results. This research establishes a foundational basis for selecting the optimal model by clarifying the performance disparities and training stability of each model in the lesion identification task.

**Figure 7:**
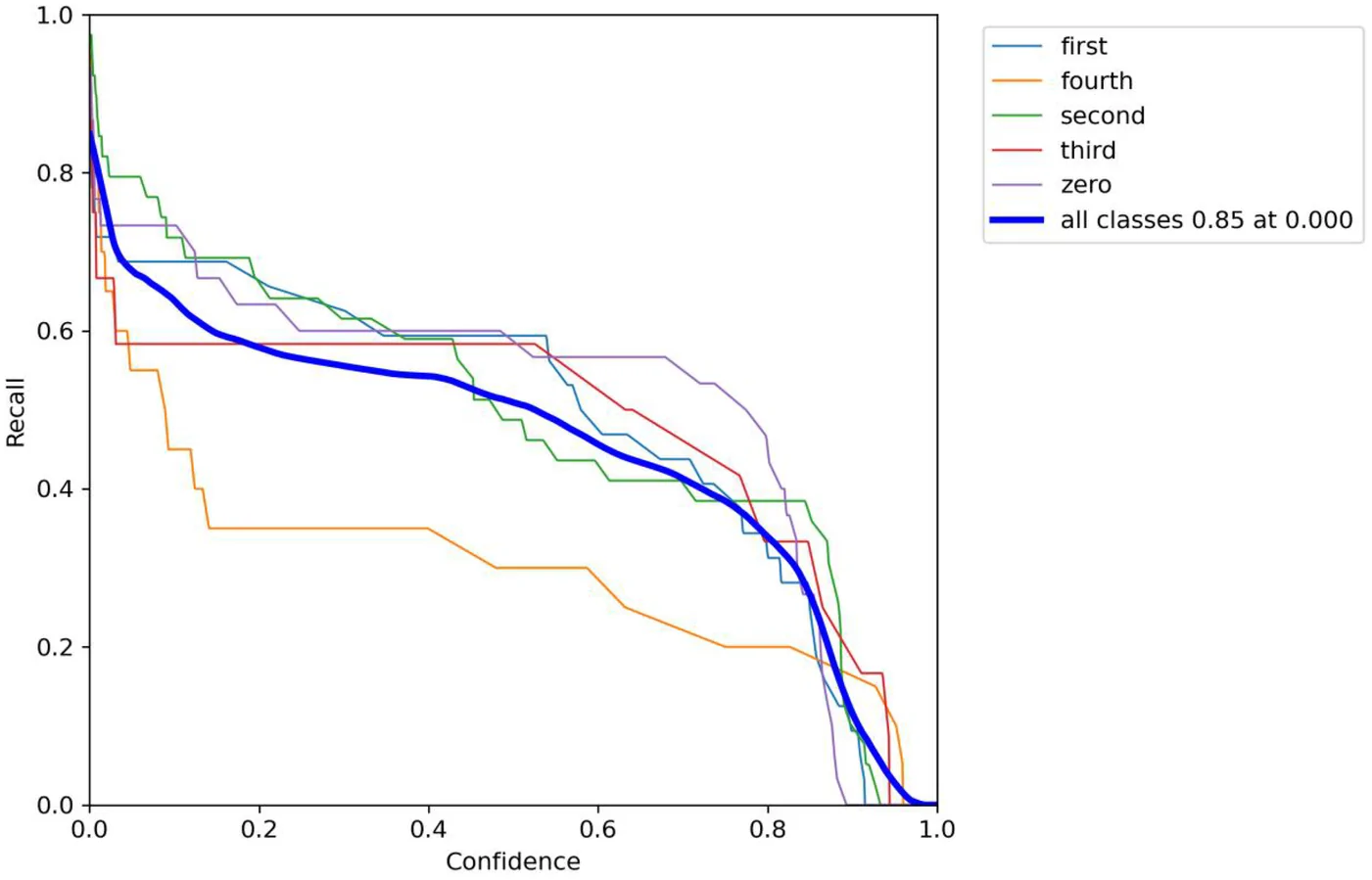
Recall for multiple models.

**Figure 8:**
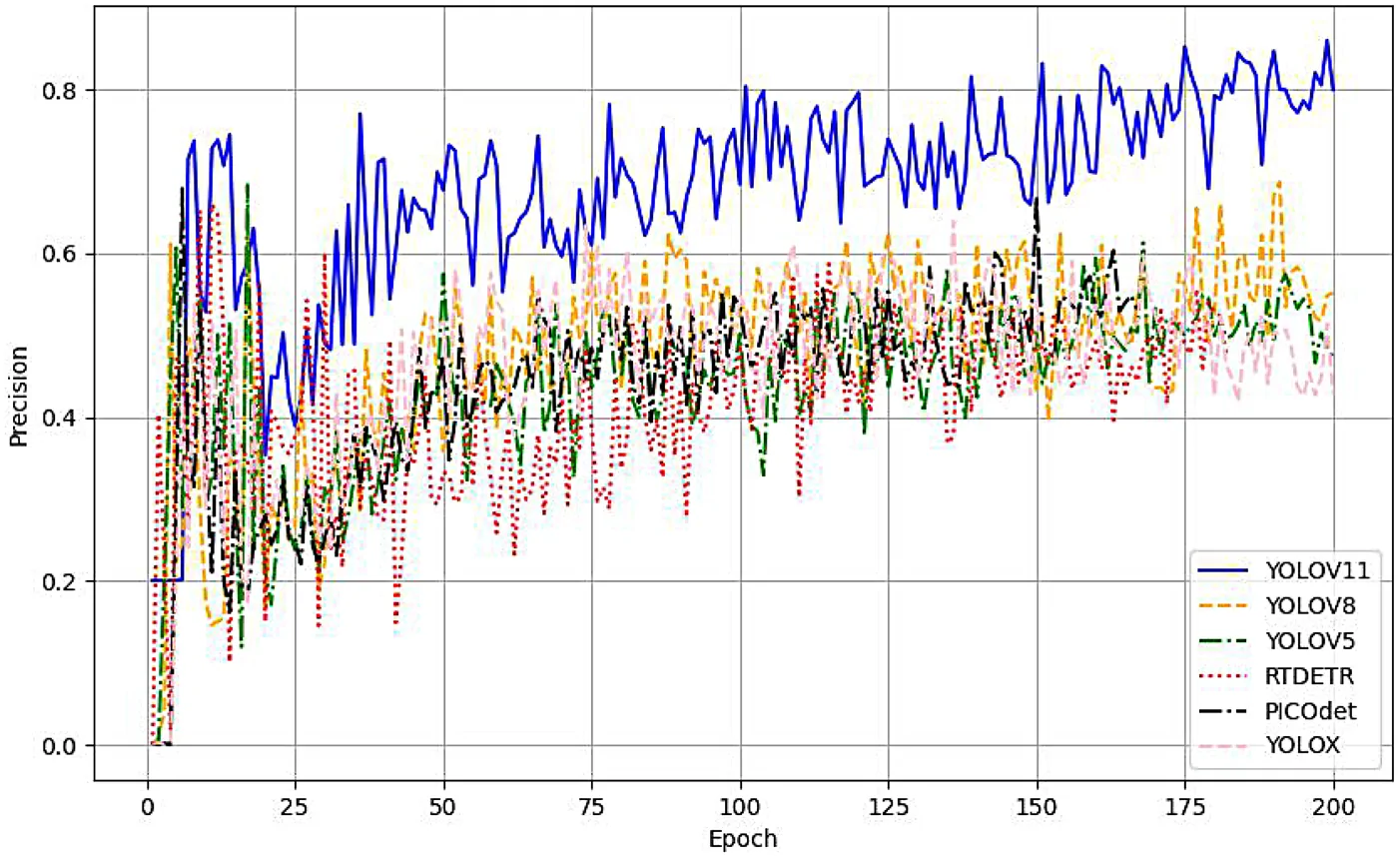
Precision for multiple models.

The YOLOv11 model surpasses previous models in recall, demonstrating superior “comprehensive detection” and training stability. The YOLOv11 model consistently demonstrates outstanding performance throughout the training cycle, as illustrated by the recall variation trend in Figure 8. During the bulk of training phases, its recall value remains consistently above 0.85; in some training intervals, it even nears 0.9. This result clearly illustrates the exceptional coverage capabilities of the YOLOv11 model for nipple lesion samples. It significantly reduces the probability of missing minor or concealed lesions by efficiently and rapidly detecting lesion samples within the dataset. This aligns with the essential clinical diagnostic need for “miss-free detection.”

The YOLOv11 model has remarkable precision and reliability in classification accuracy. Figure 9 depicts the precision curve, demonstrating that the model's precision consistently exceeded 0.85 across most training phases, reaching a maximum of 0.9 during particular training sessions. This indicates that the proportion of actual injury cases is significantly elevated among samples that YOLOv11 predicted to have nipple injuries. Its precise classification skills safeguard detection accuracy and provide reliable decision support for therapeutic interventions by significantly minimizing false positives, which arise when normal nipple regions are erroneously recognized as injuries.

**Figure 9:**
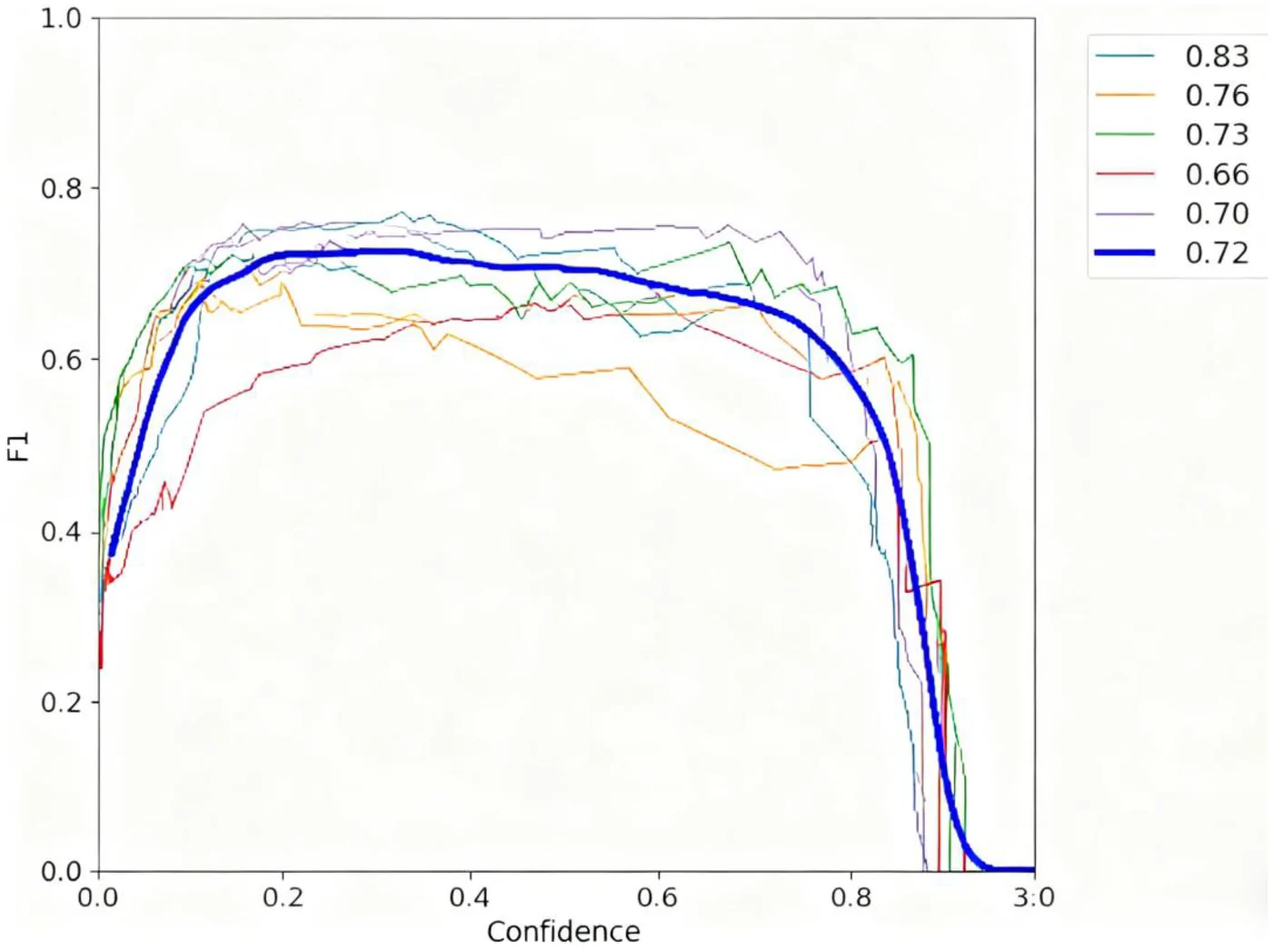
F1-confidence curve for multiple models.

The YOLOv11 model demonstrates a distinct superiority in nipple damage detection tasks when considering memory and precision. YOLOv11 surpasses benchmark models like as YOLOv8, YOLOv5, RTDETR, PICodet, and YOLOX in lesion sample coverage and detection accuracy, while also demonstrating enhanced stability during training. This presents considerable application potential and clinical translation value, rendering it a promising choice for automated identification of nipple lesions.

Figure 9 illustrates the F1-confidence curve for the nipple lesion identification task. This curve illustrates model output confidence as the independent variable and F1 score as the dependent variable, with multiple color-coded curves representing different benchmark F1 performance levels. It offers a visual depiction of the model's performance evolution when the confidence threshold varies. The overarching trend across all models exhibits a clear pattern of “swift escalation at low confidence – stable retention at medium confidence – precipitous decline at high confidence”: the F1 score ascends rapidly as confidence rises within the low-confidence threshold of 0–0.2, signifying the model's effectiveness in preemptively identifying high-confidence samples; all curves sustain relatively stable high values within the medium-confidence range of 0.2 to 0.8, reflecting the model's consistent output domain. Nevertheless, the F1 score abruptly declines to 0 when confidence approaches the exceptionally high threshold of 3.0, indicating that excessively stringent confidence criteria significantly diminish the occurrence of authentic detection results. In terms of performance stratification, the curve associated with the benchmark F1 = 0.83 exhibits optimal performance across the entire confidence interval: the range of confidence levels sustaining high performance is significantly broader than that of other curves, and its F1 value remains near 0.8 within the stable output domain. The F1 scores of the remaining curves decline progressively with the benchmark performance level, creating a consistent performance gradient aligned with the F1 tiers depicted in the figure.

This curve further validates the performance stability of high-baseline F1 models, which maintain superior detection skills across broader confidence threshold ranges. In clinical settings, this attribute provides mathematical assistance for selecting confidence thresholds tailored to varying risk preferences. Furthermore, from the perspective of confidence-performance correlation, it substantiates the technical suitability of high-F1 models for the diagnosis of nipple lesions.

Figure 10 displays the training-validation metrics of the YOLOv11 model for the classification of nipple lesions. It encompasses two categories of curves: loss functions and core performance indicators, which provide a transparent representation of the model's performance variations across time. The experiment examines the variation patterns of three loss functions: distribution fitting loss (dfl_loss), which assesses the model's ability to conform to the distribution characteristics of lesion targets; bounding box loss (box_loss), which indicates the precision of lesion center localization and bounding box regression; and classification loss (cls_loss), which measures the model's accuracy in distinguishing between nipple lesion categories. Box_loss, cls_loss, and dfl_loss exhibit a consistent decline throughout the iterations (0–200 epochs) until reaching stability, as seen by the loss curves during the training phase. This indicates that as parameters are refined, the model's proficiency in lesion categorization, regional localization, and dispersion characteristics continues to improve. The model's ability to generalize is validated by the validation phase (val) loss curve, which exhibits minor fluctuations but predominantly trends downward in accordance with the training loss. Concurrently, the model's precision, recall, mAP50, and mAP50-95 increase rapidly with the progression of iterations, attaining a stable high-value plateau after 100 epochs, as illustrated by the performance metric curves on the right. This outcome aligns with the progression of the loss function: The convergence of loss measures indicates an enhancement in model performance, while the stability of performance indicators validates YOLOv11's suitability for nipple lesion classification tasks, establishing a foundation for future clinical applications.

**Figure 10:**
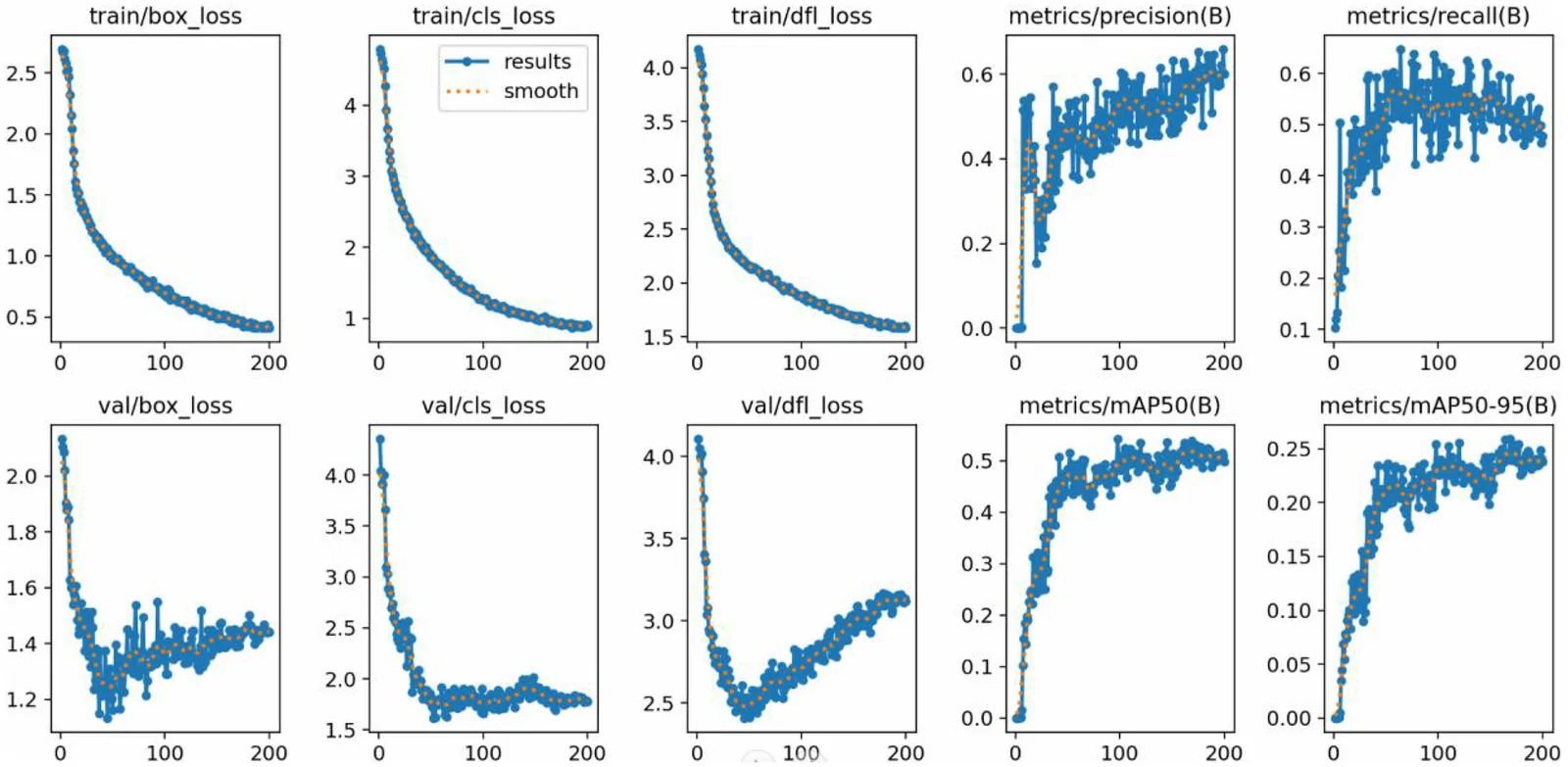
Training-validation metric curves of the YOLOv11 model in nipple injury classification task.

Figure 11 illustrates the inference output images generated by the YOLOv11 model for nipple lesion detection, showcasing detection results for six samples from the nipple region. The model identifies lesion areas using bounding boxes, each annotated with the lesion staging category (‘zero’, ‘first’, ’second’, ‘third’, ‘fourth’ corresponding to stages 0-IV) and the corresponding confidence level (0.8-1.0).

**Figure 11:**
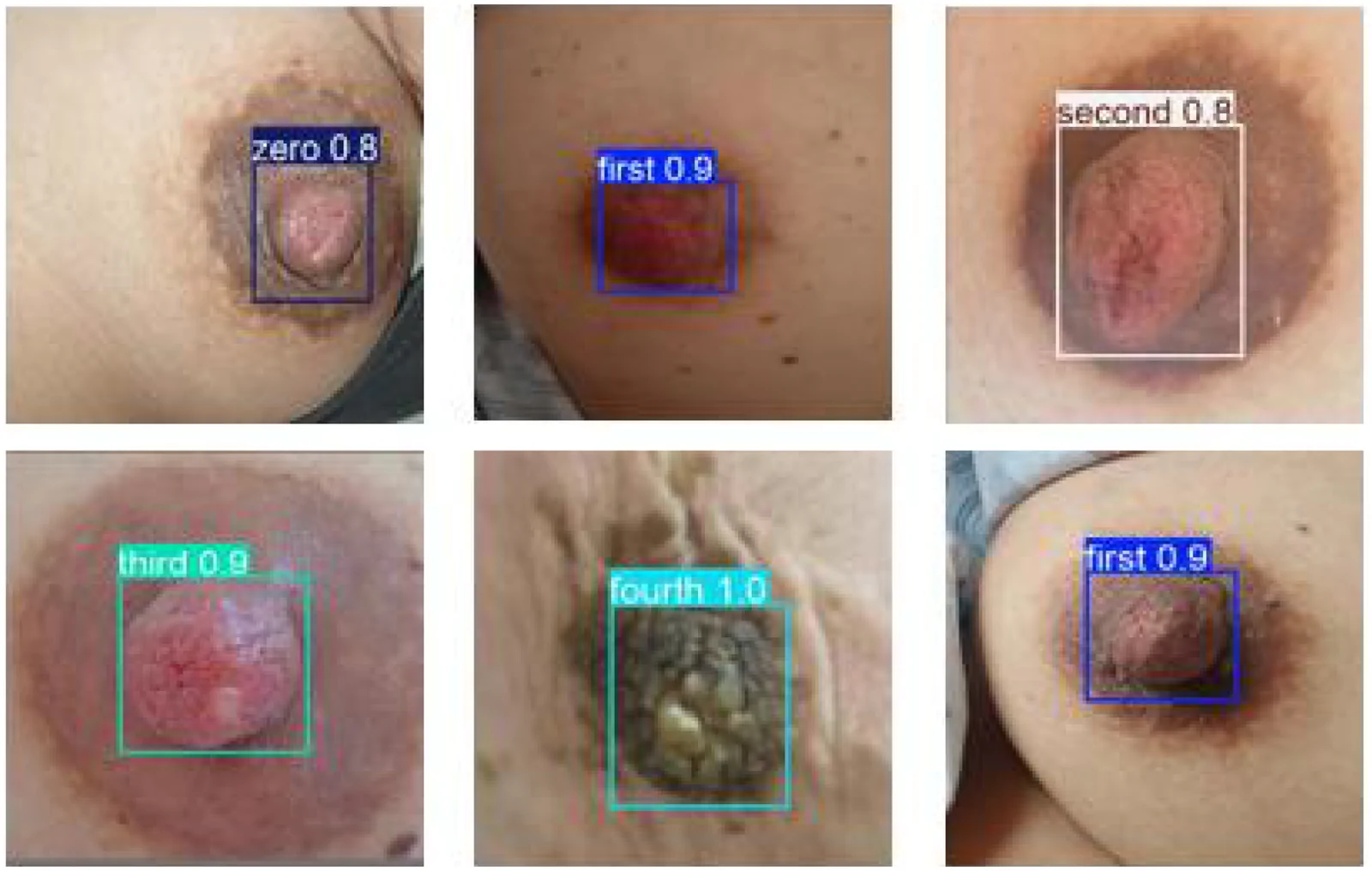
YOLOv11 nipple injury detection inference result.

The inference results illustrate YOLOv11's accurate detection and staging categorization of nipple lesions: all lesion locations are precisely included by bounding boxes, with staging labels according to the real severity of the lesions. Moreover, all detection outcomes demonstrate confidence levels of 0.8 or above, with ‘fourth’ stage lesions attaining a confidence score of 1.0, so affirming the reliability of the model's output.

This result clearly illustrates YOLOv11's technical superiority in small object recognition tasks: its advanced feature extraction architecture is tailored to the task's characteristic of a ’small proportion of nipple lesion areas.’ This, in conjunction with optimized training data, substantially improves detection and classification precision. In comparison to other YOLO series models, YOLOv11 exhibits enhanced performance in this challenge. The high-confidence detection results substantiate its potential application in automated nipple lesion assessment, offering technical assistance for accurate lesion identification in clinical settings.

## 5 Ablation study

To quantitatively identify the factors driving YOLOv11 performance, controlled ablation experiments were conducted while keeping the backbone, optimiser, and evaluation protocol fixed. Four factors were examined: (i) data augmentation strategy, (ii) input resolution, (iii) loss-function configuration, and (iv) multi-scale detection.

Quantitative ablation outcomes are summarised in Table 6 and visualised in Figure 12.

**Table 6:** Ablation results of YOLOv11 (metric: mAP@0.5).

Ablation factor	Setting	mAP@0.5
Data augmentation	No augmentation (613 images)	0.71
Data augmentation	Standard geometric/colour augmentation	0.79
Data augmentation	Stage-aware clinical augmentation	0.84
Input resolution	416 × 416	0.76
Input resolution	640 × 640 (default)	0.84
Input resolution	800 × 800	0.85
Loss configuration	Full loss (+DFL)	0.84
Loss configuration	Without DFL	0.80
Loss configuration	Box–classification imbalance	0.78
Multi-scale detection	Enabled	0.84
Multi-scale detection	Disabled	0.80

**Figure 12:**
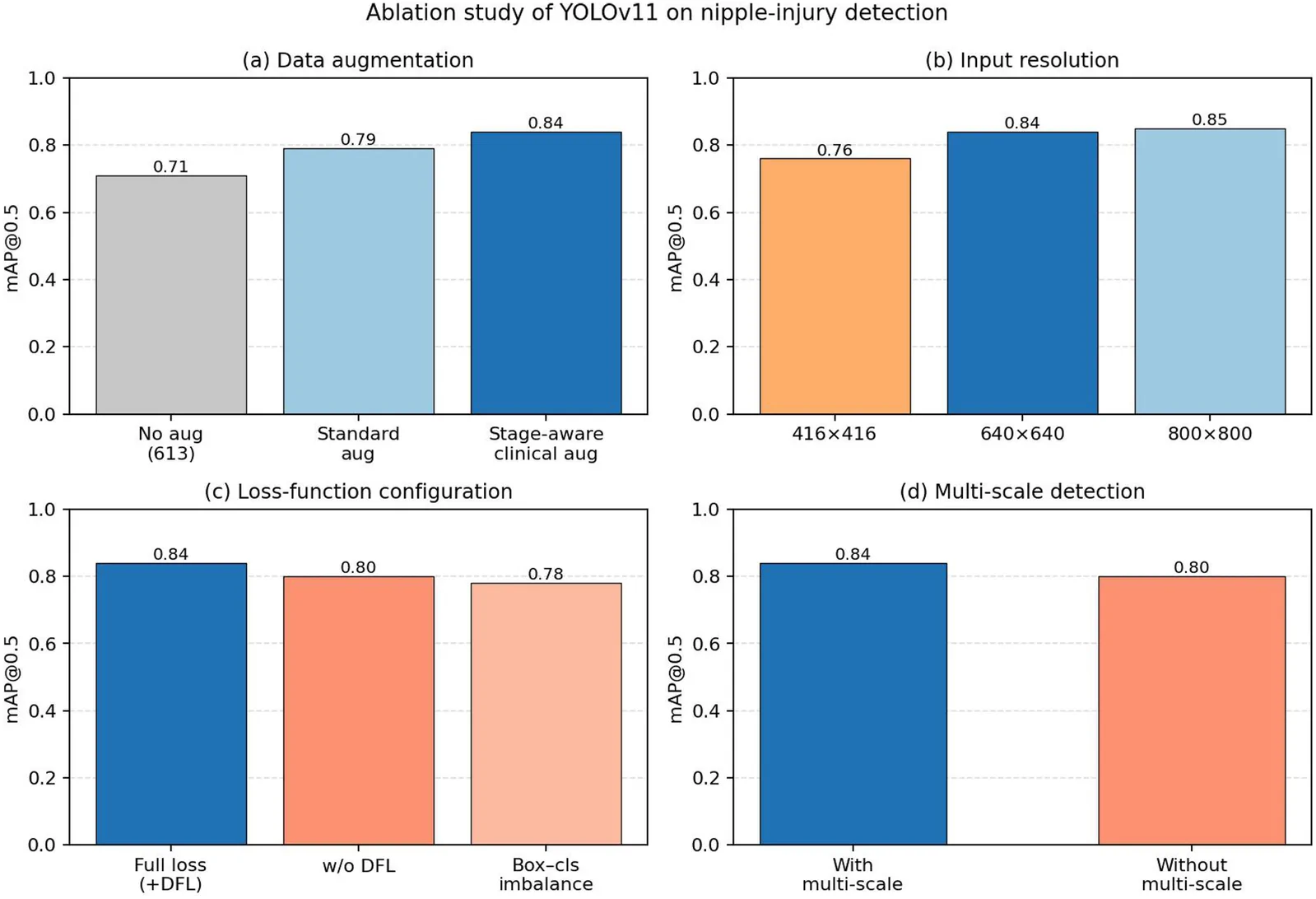
Ablation study of YOLOv11—**(a)** data augmentation, **(b)** input resolution, **(c)** loss-function configuration, and **(d)** multi-scale detection.

First, three augmentation settings were compared: no augmentation (613 original images), standard geometric/colour augmentation, and stage-aware clinical augmentation (lesion-preserving cropping + local CLAHE + class-aware sampling). Relative to the non-augmented baseline (mAP@0.5 = 0.71), standard augmentation improved mAP@0.5 to 0.79, and stage-aware clinical augmentation further improved mAP@0.5 to 0.84, accompanied by gains in recall for early-stage lesions. These results indicate that clinically motivated augmentation, rather than sample size expansion alone, is a major contributor to robustness.

Second, input resolutions of 416 × 416, 640 × 640, and 800 × 800 were evaluated. The 640 × 640 setting achieved the best accuracy–efficiency trade-off (mAP@0.5 = 0.84), whereas 416 × 416 reduced small-lesion recall and 800 × 800 yielded only marginal accuracy gains at substantially higher computational cost. Third, ablating distribution focal loss (DFL) or disrupting the default box–classification loss balance decreased localisation stability for tiny nipple targets, confirming that the compound loss configuration is necessary. Fourth, disabling multi-scale feature fusion/multi-scale training reduced mAP@0.5 by approximately 0.03–0.04, with a more pronounced drop for Stage I–II lesions.

Collectively, the ablation results show that YOLOv11’s superior performance arises from the combined contribution of architectural capacity, stage-aware data augmentation, appropriate input resolution, and multi-scale detection, rather than from any single factor in isolation.

## 6 Conclusion

This study methodically analyzed and assessed six single-stage object detection models—YOLOX, PICOdet, YOLOv5, RTDETR, YOLOv8, and YOLOv11—together with representative two-stage baselines (Faster R-CNN and Cascade R-CNN) for the diagnosis of nipple lesions. Results demonstrate that the YOLOv11 model exhibits the most advantageous overall performance among the detectors compared in this study.

The results reveal that the YOLOv11 model had enhanced overall performance in this task, attaining accuracy, recall, and mean average precision (mAP) of 0.85, 0.82, and 0.84 correspondingly, while demonstrating greater convergence rates and training stability. The model proficiently differentiates between normal (Stage 0) and any traumatic stage. An examination of the confusion matrix indicates the highest detection rate for lesions in Stage III (notable characteristics).

This research is the inaugural comprehensive experimental comparison that identifies YOLOv11 as the strongest-performing model among the single-stage and two-stage detectors compared for automated detection and staging of nipple lesions. It offers a rigorously proven, cutting-edge model solution and performance benchmark for this domain. The automated detection tool effectively converts the subjective clinical assessment method into an objective, quantitative, and reproducible analytical workflow. This offers healthcare workers real-time, consistent diagnostic assistance, thereby mitigating the danger of overlooked diagnoses and improving clinical efficiency. This tool functions as a standardized assessment approach for precisely monitoring and evaluating the therapeutic benefits of various therapies. It also possesses potential for incorporation into mobile health platforms, allowing breastfeeding women to self-assess injuries and commence early preventive therapies.

This research possesses multiple limitations. Data samples are derived from a singular center, and photos were obtained in a regulated environment, contrasting with authentic self-captured situations. Moreover, the model exclusively depends on visual information, lacking the incorporation of multimodal clinical data, including breastfeeding frequency and pain scores. The study concentrated on technical performance for validation, rather than conducting prospective clinical studies to ascertain its actual impact on breastfeeding results.

Future endeavors may focus on multi-center data validation, the integration of multimodal clinical information, and the execution of prospective efficacy evaluations to enhance the tool’s extensive utilization in practical clinical environments.

## 7 Discussion

Nipple trauma is a key factor contributing to the premature discontinuation of breastfeeding in lactating women. The early identification and precise staging are crucial for clinical intervention. Conventional techniques dependent on visual evaluation by nursing personnel exhibit significant subjectivity, low consistency, and restricted efficiency, especially in differentiating early-stage injuries from normal tissue or identifying nuanced variations across distinct stages. In recent years, deep learning-based object detection algorithms have shown considerable promise in medical picture analysis. The YOLO series of models has attained commendable outcomes in numerous lesion recognition tasks owing to their excellent real-time detection abilities. Nonetheless, regarding the specific task of nipple lesion detection—distinguished by morphological variability, diminutive size, and significant color heterogeneity—despite initial investigations by Nakamura et al. (17) into deep learning applications, no comprehensive comparison of the performance of various YOLO versions has been undertaken to ascertain the optimal model selection.

This work constitutes the inaugural thorough comparison of six prominent YOLO models (YOLOX, PICOdet, YOLOv5, RTDETR, YOLOv8, and YOLOv11) in the diagnosis and staging of nipple lesions. The results indicate that YOLOv11 exhibits exceptional performance across all critical parameters, achieving an accuracy of 0.85, a mean average precision (mAP) of 0.84, and a recall of 0.82, markedly surpassing other models. This discovery not only confirms YOLOv11's strong ability to manage small targets and intricate morphological variations but also establishes the current optimal model selection foundation for the automated, objective evaluation of nipple lesions. Beyond the single-stage family, we further compared Faster R-CNN and Cascade R-CNN as two-stage baselines under a unified IoU protocol; YOLOv11 remained superior (mAP@0.5 = 0.84 versus 0.621 and 0.470). Ablation analyses further indicated that stage-aware augmentation and multi-scale detection contribute measurably to this advantage, providing a more mechanistic explanation than architecture-only interpretations.

This work improved model generalization and robustness by standardized clinical picture acquisition techniques and meticulous data augmentation strategies. All photos were obtained using consistent equipment and settings, subsequently increasing from 613 original images to 2452 by various augmentation methods, including random flipping, color space modifications, and noise infusion. This accurately replicated intricate clinical scenarios, including variances in illumination, viewing angles, and skin tone discrepancies. This research offers systematic validation throughout the entire workflow—encompassing data construction, model training, performance comparison, and mobile deployment—unlike previous studies, such as Sen (18) et al.'s classification based on extracted wound plaques (18) and Chattree's (19) model-based contour detection and wound area/dimension analysis, which depended on manual feature extraction or single-model testing.

This study use confusion matrices to evaluate model recognition performance and confusion patterns at various phases. The model exhibits outstanding clinical reliability, with a recognition rate of 0.96 for normal nipples (Stage 0). It attains optimal recognition for Stage III lesions (0.87), signifying robust capture capabilities for lesions exhibiting different morphological characteristics. Despite misclassifications between Stage I and Stage IV lesions, likely attributable to their visual resemblance as ‘red areas’ in static images, this observation distinctly underscores a clear pathway for future improvement: enhancing model discrimination by augmenting stage-specific contrast samples, incorporating texture features, or integrating multimodal information. The model's significance encompasses therapeutic research. This methodology facilitates accurate patient staging by integrating efficacy data from Nakamura et al. (20), Wang et al. (21), Savaş et al. (22), and Shetty et al. (23). This enables the scientific assessment of treatment-stage connections, hence promoting personalized therapy.

This study effectively implements the ideal model in a mobile application, showcasing its clinical relevance and promise for real-time diagnostic assistance. Conventional assessment techniques are labor-intensive and reliant on experience, while our methodology facilitates immediate, objective staging results. This not only offers decision help for healthcare professionals but also enables lactating ladies with self-monitoring and early warning functionalities. This technique provides a more explicit clinical implementation pathway and substantial practical utility compared to earlier studies limited to model performance reports.

This study demonstrates YOLOv11's superiority among the compared single-stage and two-stage detectors for nipple-injury detection and staging through systematic comparison, two-stage benchmarking, and ablation validation. It exhibits considerable methodological benefits and potential for clinical use in data standardization, augmentation techniques, and model implementation. The study presents an effective and dependable technical solution for the assessment of intelligent nipple injuries, while also establishing a reproducible research framework for analogous medical image analysis tasks that involve small targets and significant heterogeneity.

## Data Availability

The original contributions presented in the study are included in the article/Supplementary Material, further inquiries can be directed to the corresponding author.
